# Operationalising the 20-minute neighbourhood

**DOI:** 10.1186/s12966-021-01243-3

**Published:** 2022-02-12

**Authors:** Lukar E. Thornton, Ralf-Dieter Schroers, Karen E. Lamb, Mark Daniel, Kylie Ball, Basile Chaix, Yan Kestens, Keren Best, Laura Oostenbach, Neil T. Coffee

**Affiliations:** 1 Department of Marketing, Faculty of Business and Economics, University of Antwerp, Antwerp, Belgium; 2 Institute for Physical Activity and Nutrition (IPAN), School of Exercise and Nutrition Sciences, Deakin University, Geelong, Victoria Australia; 3 Health Research Institute, University of Canberra, Canberra, Australia; 4 Melbourne School of Population and Global Health, The University of Melbourne, Parkville, Australia; 5 Department of Medicine, St Vincent’s Hospital, The University of Melbourne, Melbourne, Australia; 6 INSERM, Institut Pierre Louis d’Épidémiologie et de Santé Publique, Sorbonne Universités, Paris, France; 7 École de Santé Publique de l’Université de Montréal (ESPUM), Montreal, QC Canada; 8 Centre de recherche du CHUM (CRCHUM), Université de Montréal, Montreal, QC Canada

**Keywords:** Neighbourhood, Built environment, Urban planning, Geographic information systems (GIS), Active transport

## Abstract

**Background:**

Recent rapid growth in urban areas and the desire to create liveable neighbourhoods has brought about a renewed interest in planning for compact cities, with concepts like the 20-minute neighbourhood (20MN) becoming more popular. A 20MN broadly reflects a neighbourhood that allows residents to meet their daily (non-work) needs within a short, non-motorised, trip from home. The 20MN concept underpins the key planning strategy of Australia’s second largest city, Melbourne, however the 20MN definition has not been operationalised. This study aimed to develop and operationalise a practical definition of the 20MN and apply this to two Australian state capital cities: Melbourne (Victoria) and Adelaide (South Australia).

**Methods:**

Using the metropolitan boundaries for Melbourne and Adelaide, data were sourced for several layers related to five domains: 1) healthy food; 2) recreational resources; 3) community resources; 4) public open space; and 5) public transport. The number of layers and the access measures required for each domain differed. For example, the recreational resources domain only required a sport and fitness centre (gym) within a 1.5-km network path distance, whereas the public open space domain required a public open space within a 400-m distance along a pedestrian network and 8 ha of public open space area within a 1-km radius. Locations that met the access requirements for each of the five domains were defined as 20MNs.

**Results:**

In Melbourne 5.5% and in Adelaide 7.6% of the population were considered to reside in a 20MN. Within areas classified as residential, the median number of people per square kilometre with a 20MN in Melbourne was 6429 and the median number of dwellings per square kilometre was 3211. In Adelaide’s 20MNs, both population density (3062) and dwelling density (1440) were lower than in Melbourne.

**Conclusions:**

The challenge of operationalising a practical definition of the 20MN has been addressed by this study and applied to two Australian cities. The approach can be adapted to other contexts as a first step to assessing the presence of existing 20MNs and monitoring further implementation of this concept.

**Supplementary Information:**

The online version contains supplementary material available at 10.1186/s12966-021-01243-3.

## Background

Estimates suggest that in 2018, 4.2 billion people (55% of the world population) lived in cities and by 2050, 68% of the world’s population will live in urbanised areas [1]. Australia is witnessing a rapid population increase in its major cities [2]. Seventy-five percent of Australia’s population growth over the last 20 years occurred in state capital cities [2, 3] and further significant population growth is forecast [4].

The global transition to urban living has occurred concomitant with increases in obesity and chronic diseases related to inappropriate diet and physical inactivity [5, 6]. Understanding how urbanisation and urban design inhibit, or alternately, promote healthful lifestyles, is essential to preventing obesity and chronic disease [7, 8]. Creating liveable urban environments that facilitate improved population health presents challenges and opportunities for governments, planners, and policy-makers responsible for employment, transport, housing, the environment, community engagement, urban sprawl, education and health [3, 8–11], all of which are key indicators of liveability [12, 13].

Compact city policies seek to ensure residents have access to important everyday amenities and services without travelling far from home and without resorting to motorised transport. In theory, neighbourhoods with a wide range of local amenities, services, and transport infrastructure, should encourage greater local interaction and support more healthful choices.

### International adoption of compact city strategies

Many Asian cities are designed in a way that reflects compact cities and consequently this results in local and, especially, vertical living [14, 15]. Portland, USA, initially promoted their compact city concept within the framework of a “20-minute neighbourhood” (20MN) [16, 17]. A 20MN was defined as *“a place with convenient, safe, and pedestrian-oriented access to the places people need to go to and the services people use nearly every day: transit, shopping, healthy food, school, parks, and social activities”* (p.4, [16]) and noted that the 20MN term *“is not intended to convey a specific metric*” (p.4 [16]). Recently, cities such as Paris, France [18], Edinburgh, UK [19], Seattle, USA [20], and the Flanders region of Belgium [21] have put forward similar concepts. In England, an emphasis is being placed on creating 20MNs with benefits stated to extend to the economy, environment, health, as well as social benefits such as safety and inclusiveness [22].

### What is happening in Melbourne?

Increasingly, Australian city planners are examining opportunities to create compact localised environments [23–35]. In Melbourne, Australia’s second largest city, the most recent planning strategy, Plan Melbourne, proposed an agenda to manage urban growth and meet Melbourne’s future environmental, population, housing and employment needs [26]. A key component underpinning Plan Melbourne was the promotion of 20MNs that allowed people to access amenities and services near their home promoting and enabling healthful local living [24, 26].

Table 1 presents an overview of the four key policy documents relating to Melbourne’s 20MN strategy with iterations published in 2014, 2015, 2017 and 2019. This table identifies how key aspects of the Melbourne 20MN have evolved and changed over this timeframe. When the 20MN concept was first introduced in the 2014 Plan Melbourne planning strategy, it ambitiously stated that *“20-minute neighbourhoods are places where you have access to local shops, schools, parks, jobs and a range of community services within a 20-minute trip from your front door”* [27] (page numbers for the quoted text throughout are provided in Table 1). The most recent version (2019) states *“The 20-minute neighbourhood is all about ‘living locally’ – giving people the ability to meet most of their daily needs within a 20-minute walk from home, with access to safe cycling and local transport options”* [24].

**Table 1:** The variations in the 20-minute neighbourhood concept across different iterations of Plan Melbourne

Document/year	Definition	Features of a 20MN	How is 20 minutes defined?	Projected benefits
Plan Melbourne 2014 [27] ^a^	20-minute neighbourhoods are places where you have access to local shops, schools, parks, jobs and a range of community services within a 20-minute trip from your front door. (Page 11) Plan Melbourne aims to create a city of 20-minute neighbourhoods where people have safe and convenient access to the goods and services they need for daily life within 20 minutes of where they live, travelling by foot, bicycle or public transport. (Page 117)	Shops, cafés and restaurants, early-years centres, primary and secondary schools, parks and sporting fields, medical centres and public transport. (Page 114) Playground, parks and greenery, cycling and walking, community centres, employment centres, local bus services, public transport to key centres, shared community open space including food growing, local shops and services, day care centres and schools, local gathering places. (Figure 14, Page 115) This includes a variety of housing choices, shops and commercial services, schools, parks and recreation opportunities and good walking and bicycle infrastructure. (Page 117)	Within 20 minutes of where they live, travelling by foot, bicycle or public transport. (Page 117)	20-minute neighbourhoods help improve health and wellbeing, reduce travel costs and traffic congestion, and reduce vehicle emissions. They also create opportunities to provide a greater diversity of housing choices close to where goods and services are located. (Page 114)
Plan Melbourne refresh - Discussion paper 2015 [25] ^a^	The ability to meet your everyday (non-work) needs locally, primarily within a 20-minute walk. (Fig. 1, Page 22) This concept is about living locally not specifically working locally. Although more local jobs are a consequence of more services and facilities locally. (Fig. 1, Page 22)	Everyday needs: This may include facilities such as schools, shops, meeting places, open space, cafés, doctors, childcare and access to public transport (Fig. 1, Page 22)	Primarily within a 20-minute walk (Figure 1, Page 22) It is indicated that the 20 minutes equates to 1–1.5 km (Figure 2, Page 22) Additionally, the within 20 minute definition is further expanded on by stating “This is included as it is important to outline that many areas will have access below 20-minutes’ walk and lower distances to services should be encouraged” (Figure 1, Page 22)	Benefits include: • Improved health (by encouraging physical activity like walking and cycling) • Less need to travel long distances by car which reduces household travel costs • Less greenhouse gas emissions (and pollution) • Lower major infrastructure costs (by making best use of existing infrastructure) • Better sense of place and the encouragement of vibrant, convenient and safe neighbourhoods • Population growth is accommodated with more housing choice in locations with better access to services • Enhanced community and social equity benefits such as better design for the elderly, the young and parents, and more interactions living and meeting locally. (Page 21)
Plan Melbourne 2017–2050 [26] ^b^	The 20-minute neighbourhood is all about ‘living locally’—giving people the ability to meet most of their everyday needs within a 20-minute walk, cycle or local public transport trip of their home. (Page 98) A 20-minute neighbourhood must: • be safe, accessible and well connected for pedestrians and cyclists to optimise active transport • offer high-quality public realm and open space • provide services and destinations that support local living • facilitate access to quality public transport that connects people to jobs and higher-order services • deliver housing/population at densities that make local services and transport viable • facilitate thriving local economies. (Page 98)	Neighbourhood activity centres are an integral part of the city’s vibrant community life and critical to the creation of 20-minute neighbourhoods. These high streets and specialised strips of shops, cafés, small supermarkets, service businesses, community services and public spaces serve the needs of the surrounding community and provide a focus not only for local jobs but also for social interaction and community participation. (Page 99) Local shopping centres, local health facilities and services, local schools, lifelong learning opportunities, local playgrounds and parks, green streets and spaces, community gardens, sport and recreational facilities, safe streets and spaces, affordable housing options, ability to age in place, housing diversity, walkability, safe cycling networks, local public transport, well connected to public transport, jobs and services within the region, local employment opportunities. (Figure 12, Page 99) Due to the specialised and diverse nature of many people’s work, access to employment will often be outside the 20-minute neighbourhood. (Page 99)	Within a 20-minute journey from home by walking, cycling, riding or local public transport. (Figure 12, Page 99) Within a 20-minute walk, cycle or local public transport trip of their home. (Page 98)	If 20-minute neighbourhoods existed across Melbourne, it could reduce travel by nine million passenger kilometres and cut Melbourne’s daily greenhouse gas emissions by more than 370,000 tonnes. (Page 98) A 20-minute neighbourhood can create a more cohesive and inclusive community with a vibrant local economy— reducing social exclusion, improving health and wellbeing, promoting a sense of place, reducing travel costs and traffic congestion, and reducing carbon emissions across the city as a whole. (Page 99)
20-Minute Neighbourhoods – Creating a more liveable Melbourne 2019 [24] ^b^	The 20-minute neighbourhood is all about ‘living locally’ – giving people the ability to meet most of their daily needs within a 20-minute walk from home, with access to safe cycling and local transport options. (Page 22) A 20-minute neighbourhood must: • be safe, accessible and well connected for pedestrians and cyclists to optimise active transport • offer high-quality public realm and open space • provide services and destinations that support local living • facilitate access to quality public transport that connects people to jobs and higher-order services • deliver housing/population at densities that make local services and transport viable • facilitate thriving local economies. (Page 24)	Local shopping centres, local health facilities and services, local schools, lifelong learning opportunities, local playgrounds and parks, green streets and spaces, community gardens, sport and recreation facilities, safe streets and spaces, affordable housing options, ability to age in place, housing diversity, walkability, safe cycling networks, local public transport, well connected to public transport, jobs and services within the region, local employment opportunities. (Fig. 1, Page 24)	…within a 20-minute walk from home, with access to safe cycling and local transport options. (Page 22) This 20-minute journey represents an 800 m walk from home to a destination, and back again. (Page 25) 800 m has been adopted as the spatial accessibility measure of a walkable neighbourhood. This distance should be used as a guide only, as there are many factors that influence people’s ability, or desire, to walk. (Page 25) While cycling and local transport provide people with alternative active travel options to walking, these modes do not extend neighbourhoods, or access to 20-minute neighbourhood features beyond walkable catchments of 800 m. (Page 25)	Benefits of a walkable neighbourhood: • can halve household transport costs • enhances sense of community and social cohesion • walking infrastructure can provide a higher return than rail or road • support health, infrastructure and environmental savings to Victorian economy • walking infrastructure delivers $13 benefit for every $1 spent • alleviates pressure on Melbourne’s transport • increases retail trading by up to 40% • improved health and wellbeing • supports passive surveillance increasing safety • helps reduce pollution and CO2 emissions (Pages 10–11) Research suggests that this approach to planning, *(*i.e.*, building walkable compact places)*, has multiple benefits, including improved public health (mental and physical), increased safety and stronger social connections. It also reduces emissions, lowers household costs and increases environmental, economic and social sustainability. (Page 23) If 20-minute neighbourhoods existed across Melbourne, they could reduce travel by nine million passenger kilometres and cut Melbourne’s daily greenhouse gas emission by more than 370,000 tonnes. (Page 23) 20-minute neighbourhoods are the way we can think and act locally to resolve global challenges, such as reducing emissions and creating more sustainable ways of living. (Page 23)

^a^ This work is made available under the terms of the Creative Commons Attribution 3.0 Australia license

^b^ This work is licensed under a Creative Commons Attribution 4.0 International license

Apart from employment opportunities, amenities and services considered as constituting everyday needs have remained relatively stable across the various iterations of Plan Melbourne (Table 1). These have included amenities and services related to retail (with specific reference among others to food retail such as small supermarkets and cafés), education, open space, sports facilities, community services, health services, and public transport. Access to safe and well-connected pedestrian and cycling infrastructure has also been a common element.

Less consistent in Plan Melbourne has been the definition of what constitutes 20 minutes from the perspective of travel mode and what distance this equates to. Originally in 2014, this was posed as *“within 20 minutes of where they live, travelling by foot, bicycle or public transport”* [27], while the 2015 version refined this to *“primarily within a 20-minute walk”* with an estimated distance of 1 to 1.5 km [25]. The updated strategy that followed in 2017 stated *“within a 20-minute journey from home by walking, cycling, riding or local public transport”* [26], although it was unclear what *“riding”* referred to given *“cycling”* preceded it separately. It is perhaps not surprising that the 2019 update refined this once again to just include walking and this time acknowledged the benefit of access to other modes using the following statement: “*within a 20-minute walk from home with access to safe cycling and local transport options”* [24]. Importantly, it is stated that *“this 20-minute journey represents an 800m walk from home to a destination, and back again”* [24]. Two interesting points are of note here. First, instead of features being within 20 minutes, the wording is suggestive that these are now effectively within 10 minutes from home factoring in a return journey. The second point of interest is the emphasis on walking noting that *“while cycling and local transport provide people with alternative active travel options to walking, these modes do not extend neighbourhoods, or access to 20-minute neighbourhood features beyond walkable catchments of 800m”* [24].

### Problem statement and objective

Without a clear conceptualisation and operationalisation of a 20MN, it is impossible to properly implement a 20MN, much less evaluate the benefits. This study sought to develop and operationalise a practical definition of the 20MN concept. The method proposed can be utilised elsewhere and modified by adding/removing spatial data layers that represent amenities and services and altering the measures of access (e.g., by distance and mode of travel) assuming each decision rule is rationalised. Flexibility in allowing the approach to be tailored ensures it can remain relevant to different populations, contexts, and policy environments.

## Methods

### Setting

With the intense policy focus on 20MNs in Melbourne (state capital of Victoria, Australia), we chose this city as the basis for our 20MN measure. To demonstrate how the measure could be tailored and applied to a different setting, the Australian city of Adelaide (state capital of South Australia) was chosen for comparison purposes. Both the Victorian [24–27] and South Australian [29] state governments have neighbourhood design and urban renewal policies yet the two state capitals themselves differ substantially in terms of population, urban sprawl, and transportation infrastructure. The 30 Year Plan for Greater Adelaide [29], while not explicitly invoking the 20MN, refers to transit-oriented developments that support walkable and connected communities*.*

### Spatial extent

**Fig. 1:**
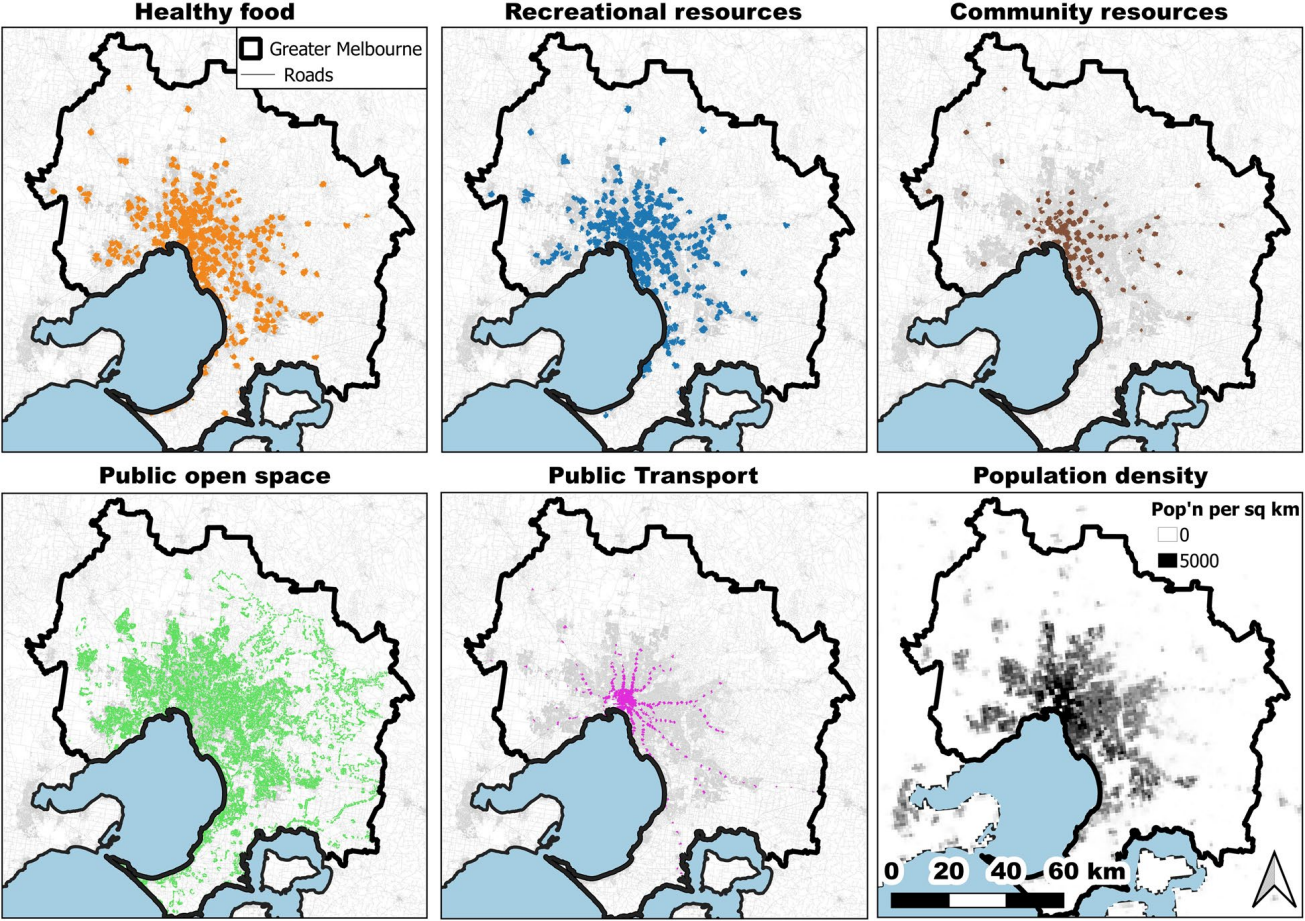
Areas in Melbourne with access to the healthy food, recreational resources, community resources, public open space, and public transport domains and a population density layer depicted by population density grid [37]

**Fig. 2:**
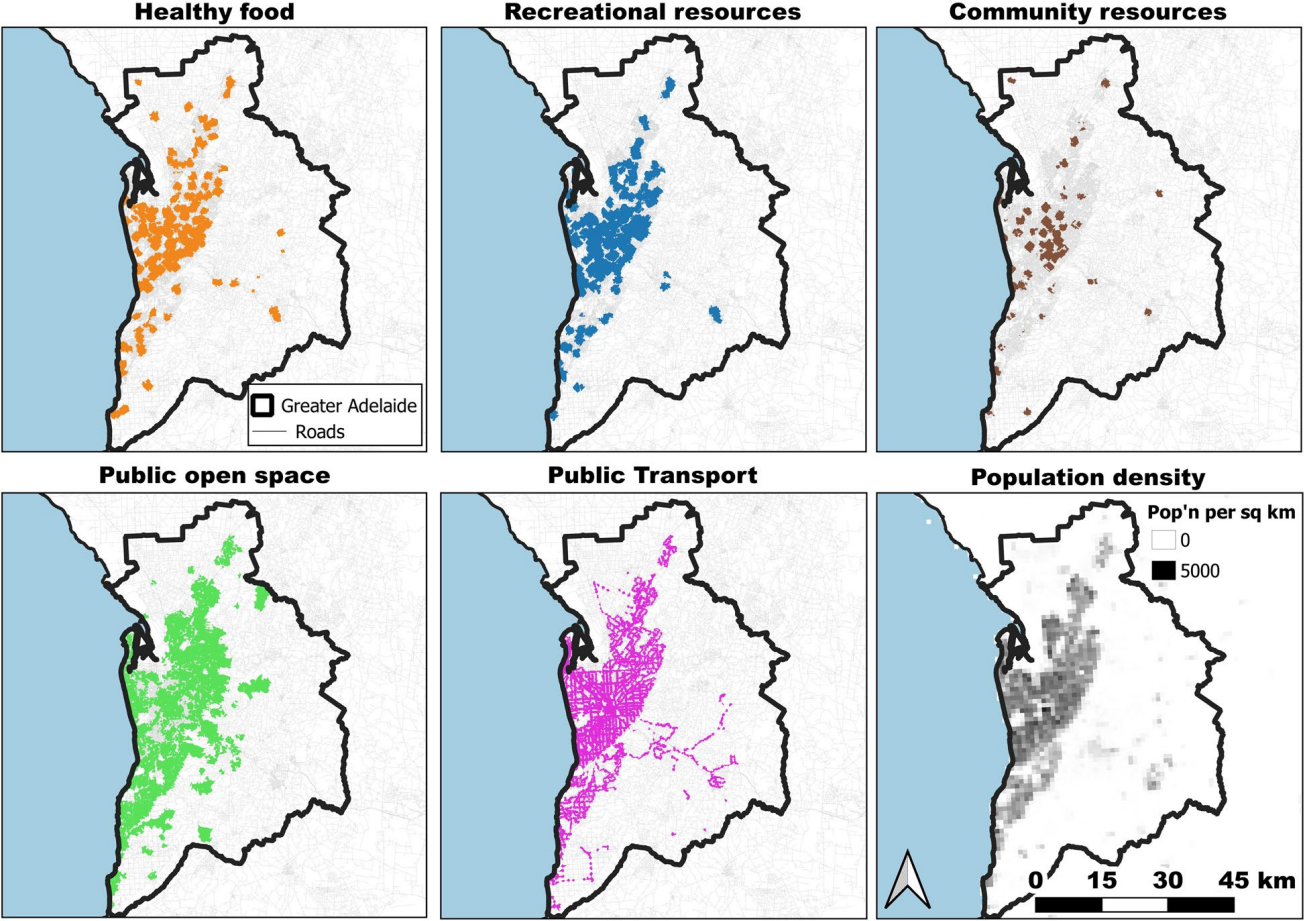
Areas in Adelaide with access to the healthy food, recreational resources, community resources, public open space, and public transport domains and a population density layer depicted by population density grid [37]

The spatial extent of the metropolitan Melbourne and Adelaide regions is represented by the 2016 Australian Bureau of Statistics Greater Capital City Statistical Areas [36] (Fig. 1, Melbourne, and Fig. 2, Adelaide). The Greater Capital City Statistical Areas represent the functional extent of the Australian State and Territory capital cities, capturing most of the commuting population and the labour markets of each capital city. They are not a marker of the edge of the city, but instead include the population who regularly socialise, shop, or work within the city, and include small towns and rural areas surrounding the urban core of the city.

### Defining the attributes of the 20-minute neighbourhood

Drawing on Plan Melbourne’s various definitions of the 20MN [24–27], the Portland Plan [16, 17], literature related to urban liveability indices (e.g. [8, 11, 12]), and our collective knowledge of this field, we proposed five spatial data domains required for a 20MN: 1) healthy food; 2) recreational resources; 3) community resources; 4) public open space; and 5) public transport. Further details of the attributes used and the inclusion rationale for each are outlined in Table 2 and in the text below.

**Table 2:** The domains, attributes, rationale for inclusion and access measure definition for features of a 20MN

Domain	Attributes	Rationale for attribute selection	Access measure
Healthy food	- Large supermarket (Coles or Woolworths) - Smaller supermarket (e.g. Aldi, IGA, Woolworths Metro, Foodland) - Fruit and vegetable store/market	The healthy food criteria were met if a resident could access a large supermarket which provides a large selection of healthy foods at affordable prices or, alternatively, access to both a smaller supermarket and a greengrocer which combined would provide opportunities to source both core household food items and healthy food products, especially fresh fruits and vegetables.	A household must meet one of the following criteria: i) A large supermarket within a 1.5-km pedestrian network distance from home OR ii) A smaller supermarket AND greengrocer (i.e. fruit and vegetable store/market) within a 1.5-km pedestrian network distance from home.
Recreational resources	- Gym	The presence of a gym (commercial or public) was used as the indicator of access to recreational facilities, recognising that many gyms offer a range of fitness options and cater to many age groups.	To meet this criterion, a household must have access to a gym within a 1.5-km pedestrian network distance from home.
Community resources	- Primary school - General practitioner (GP) - Pharmacy - Library - Post office - Café	The community resource criteria were met if an area had each of the following: 1. Primary school: School for young children (school years prep to grade 6 servicing children 5–12 years of age). 2. General practitioner: Provision of health services, particularly for elderly. 3. Pharmacy: Provision of health services, particularly for elderly. 4. Library: Learning opportunities and access to internet, particularly for children, the elderly and the socioeconomically disadvantaged. 5. Post office: Provider of government, financial and other ancillary services. 6. Café: Opportunities for social engagement.	To meet this criterion, a household must have access to each of the six community resource attributes within a 1.5-km pedestrian network distance from home.
Public open space - Melbourne	For the 400 m distance measure: public open space excluding small local links For the 8 ha within 1 km measure, publicly accessible: - Natural and semi-natural open space - Parks and gardens - Conservation reserves - Recreation corridor - Sports fields and organised recreation areas	Two measures were used: i) households within 400 m distance along a pedestrian network to public open space and ii) 8 ha of public open space within a 1-km radius. Both access measures were informed by the Victorian Planning Provisions which outline (amongst other clauses) that 95% of all dwellings should be within 400 m of a safe walking distance to local parks and have access to active open space of at least 8 ha in area within 1 km. (Standard C13 - VPP-56.05-2 Public Open Space Provision Objectives http://planningschemes.dpcd.vic.gov.au/schemes/combined-ordinances/VPPs_All_Clauses.pdf) For the open space features chosen for the 8 ha within 1 km measure, we used a Euclidean distance and included only open space where people could freely visit (i.e. only publicly accessible spaces) for sport, recreation, or relaxation/restoration purposes.	To meet this criterion, a household must have access to the following: i) Access to public open space within 400-m pedestrian network distance from home* AND ii) ≥8 ha of public open space within 1 km from home * This data source provides a measure of households within 400 m of public open space with access points defined at 30-m intervals along public open space boundaries, where direct access from roads or trails are available [38]. It also includes a road network to which the custodians added thousands of informal pedestrian links. Therefore, it provides a highly accurate representation of pedestrian access.
Public open space - Adelaide	Publicly accessible open space areas categorised within the data source as: - Botanic garden - Coastal - Conservation area - Conservation park - Linear - Linear park - Link - National park - POS - Recreation park - Sport - Wetland	Two measures were used: i) households within 400 m distance along a pedestrian network to public open space and ii) 8 ha of public open space within a 1-km radius.	To meet this criterion, a household must have access to the following: i) Access to public open space (park access points spaced every 50 m) within 400 m from home AND ii) ≥8 ha of public open space within 1 km from home
Public transport - Melbourne	- Bus stop - Tram stop - Train station	The three primary modes of public transport in Melbourne were selected for this measure. Buses and trams facilitate more localised travel within areas (and sometimes across areas), while trains facilitate longer journeys such as to and from the Melbourne central business district for work. Access measures for each mode were informed by past literature on public transport accessibility [39–41]. It was decided that within a 5-km radius centred around the central business district (with the General Post Office (GPO) as centre point), access buffers had to represent availability of either tram, bus, or train. This was factoring in that 5 km was a more than reasonable distance to assume that people who needed access to the city could do so via bus or tram without a major time burden or use alternative non-motorised forms of transport such as walking and cycling. The transport network beyond the 5 km range was modelled enforcing train availability, i.e. train stations had to be accessible in combination with either a bus stop or tram stop. This rule reflects that those in Melbourne have on average a commute distance of over 15 km [42] and trains are often the quickest mode of public transport over longer distances.	Within 5 km of the GPO: To meet this criterion, a household must have access to: i) A bus stop within a 400-m pedestrian network distance from home. OR ii) A tram stop within a 600-m pedestrian network distance from home. OR iii) A train station within an 800-m pedestrian network distance from home. Further than 5 km from the GPO: To meet this criterion, a household must have access to: i) A train station within an 800-m pedestrian network distance from home. AND either ii) A bus stop within a 400-m pedestrian network distance from home. OR iii) A tram stop within a 600-m pedestrian network distance from home.
Public transport - Adelaide	- Bus stop including O-Bahn (guided busway) - Tram stop - Train station	The three primary modes of public transport were included using the same rationale for the distance to these modes as outlined for Melbourne above. Note, a separate measure for further than 5 km from the central business district was not undertaken in Adelaide given the emphasis on different public transport modes in this city and the smaller spatial extent.	To meet this criterion, a household must have access to: i) A bus or O-Bahn stop within a 400-m pedestrian network distance from home. OR ii) A tram stop within a 600-m pedestrian network distance from home. OR iii) A train station within an 800-m pedestrian network distance from home.

### Defining the pedestrian network layer

A pedestrian network layer was created for each city which allowed for the calculation of pedestrian network distances. A pedestrian network distance differs from a road network distance by including paths that vehicles cannot use (e.g. pedestrian alleyways that link two streets) and excluding network paths accessible to cars but not pedestrians (e.g. freeways) [43]. Vehicle based one-way restrictions on streets were also removed to allow pedestrian movement in either direction, whilst tunnels and overpasses were included in the network model so as to distinguish them from crossroad intersections. For Melbourne, VicMap Transport data were used to create this layer and, in Adelaide, Statewide Road Network data.

### Data sources

Data were sourced from a combination of government and commercial sources either publicly available or accessible upon request. Some datasets were available at a national level, meaning the same data sources could be used for both Melbourne and Adelaide (e.g., general practitioners (GPs) and pharmacies), whilst others were state or city specific (e.g., public open space). For full data source details see Additional File 1.

A further comment on our featuring of both a primary city (Melbourne) and a comparison city (Adelaide) is that a great deal of spatial data representing natural and built environments in Australia is based at a state or even at more local levels rather than being available nationally in a consistent format. Thus, the use of a comparison city provides an opportunity to test the applicability of our 20MN concept in a different context where the data sources vary, further demonstrating the generalisability of the approach.

### Defining accessibility for each layer

All layers were processed using ArcGIS v10.5 [44] and access to each was defined as per Table 2. For most measures, a pedestrian network service area at a specified distance was created using the feature as the starting point noting that there were no one-way restrictions within our network layer. Address points within this service area were therefore considered to have access to this feature. For most features, a 1.5-km distance was used and is consistent with common definitions used in walkability studies which equate a 5-minute walk to 400 m [45]. For public transport, accessibility measures were informed by the literature and altered based on published estimates of usual distances that people walk to different transport modes [39–41].

The access measure used for open space considered both access to public open space within a 400-m network distance and at least 8 ha of public open space area within a 1-km radius. These access metrics were consistent with the Melbourne planning guideline recommendations for open space (see Table 2). The comprehensive Victorian Planning Authority Metropolitan Open Space Network walkable catchment layer [38] was used to determine if households were located within 400 m of open space in Melbourne with open space access points within this dataset specified by the provider at 30-m intervals. For Adelaide, data were sourced from a previous study [46] and 400-m pedestrian network service areas were created along park border points (50-m spacing). In both cities, the selected open space features outlined in Table 2 were rasterised to a 10-m × 10-m grid (cell defined as open space: 0 = no; 1 = yes). A count of open space cells within a 1-km radius (circular radius using focal statistics) around each individual cell was undertaken. A minimum count of 800 cells classified as open space was required to represent access to at least 8-ha open-space area within a 1-km radius. The method resulted in a continuous surface (10-m grid) representation of open space access across both Melbourne and Adelaide. This approach avoids some of the complex issues associated with measuring open space access [47].

### Combining layers to create domains

As detailed in Table 2, for each of the five 20MN domains (healthy food, recreational resources, community resources, public open space, public transport), several criteria had to be met for households to be defined as having access to this domain, with the exception of the recreational resources domain, which required access to a gym only. The healthy food domain required access to either a large supermarket *or* both a smaller supermarket and a greengrocer (greengrocers defined as fruit and vegetable stores). The community resources domain required access to each of the six layers (i.e., primary school, general practitioner, pharmacy, library, post office, and café). The public open space domain required access to public open space within 400 m and at least 8 ha of open space within 1 km. Finally, the public transport domain in Melbourne required access to any public transport mode for households located within a 5-km radius of the city centre (defined by the location of the General Post Office) or access to a train station *and* either a bus or tram for those beyond 5 km (see Table 2 for rationale). In Adelaide, the public transport domain required access to any of the three specified modes of transport.

Using the community resources domain as an example, determining if access to the domain criteria had been met involved overlaying the services areas of the six individual layers and extracting the intersection of the six layers. The spatial distribution of areas considered to meet the access criteria for each domain is presented in Fig. 1 for Melbourne and Fig. 2 for Adelaide.

### Creating the final 20MN layer

**Fig. 3:**
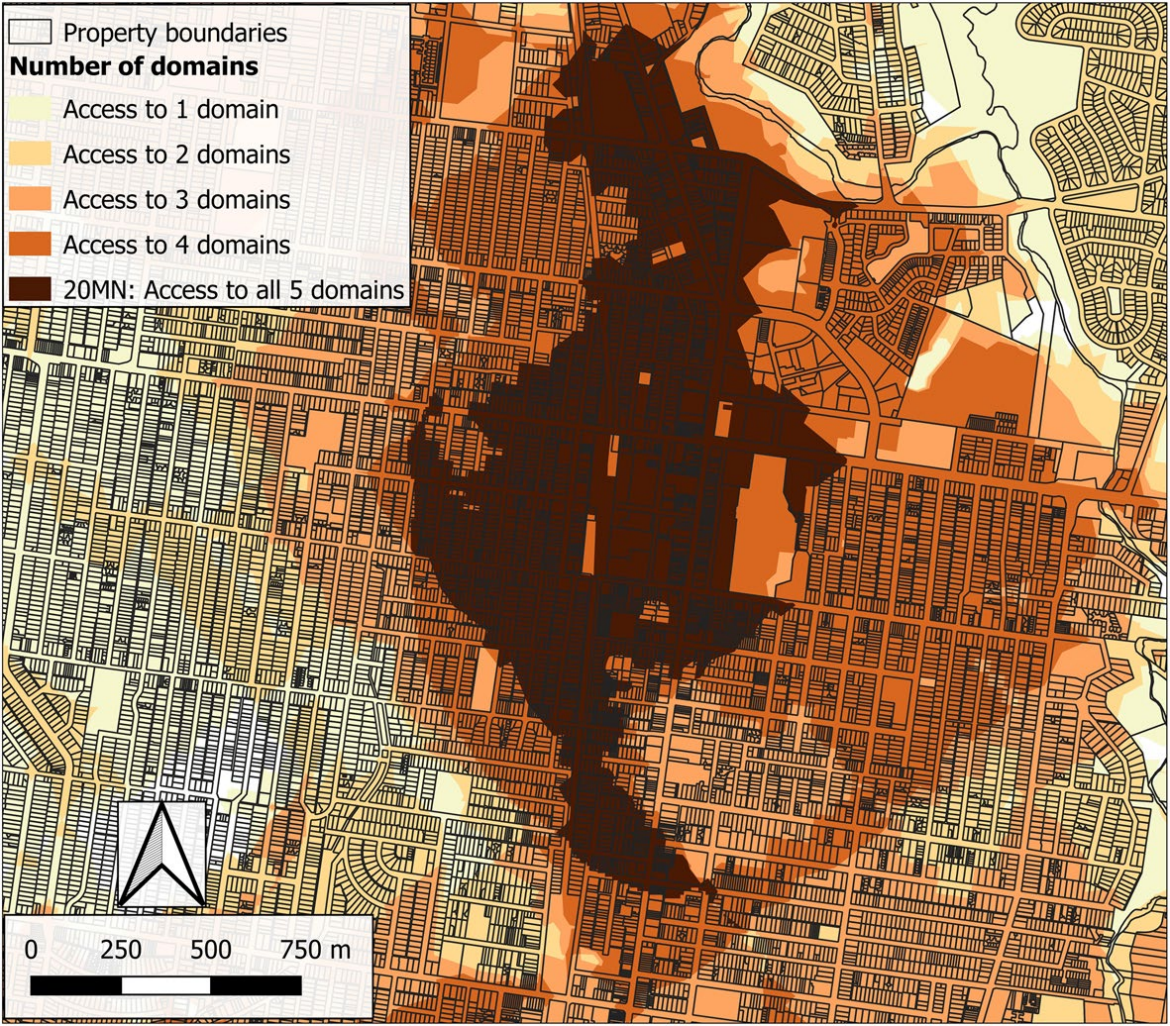
Intersecting areas of domain access

Once the five domains were generated, they were overlayed, and the count of intersecting areas calculated (Fig. 3). Areas where all five domains intersected were considered as consistent with the 20MN concept as they met the access criteria for each domain (healthy food, recreational resources, community resources, public open space, and public transport). This approach allows features to be dispersed in different directions around address points rather than clustered in a single activity centre.

**Fig. 4:**
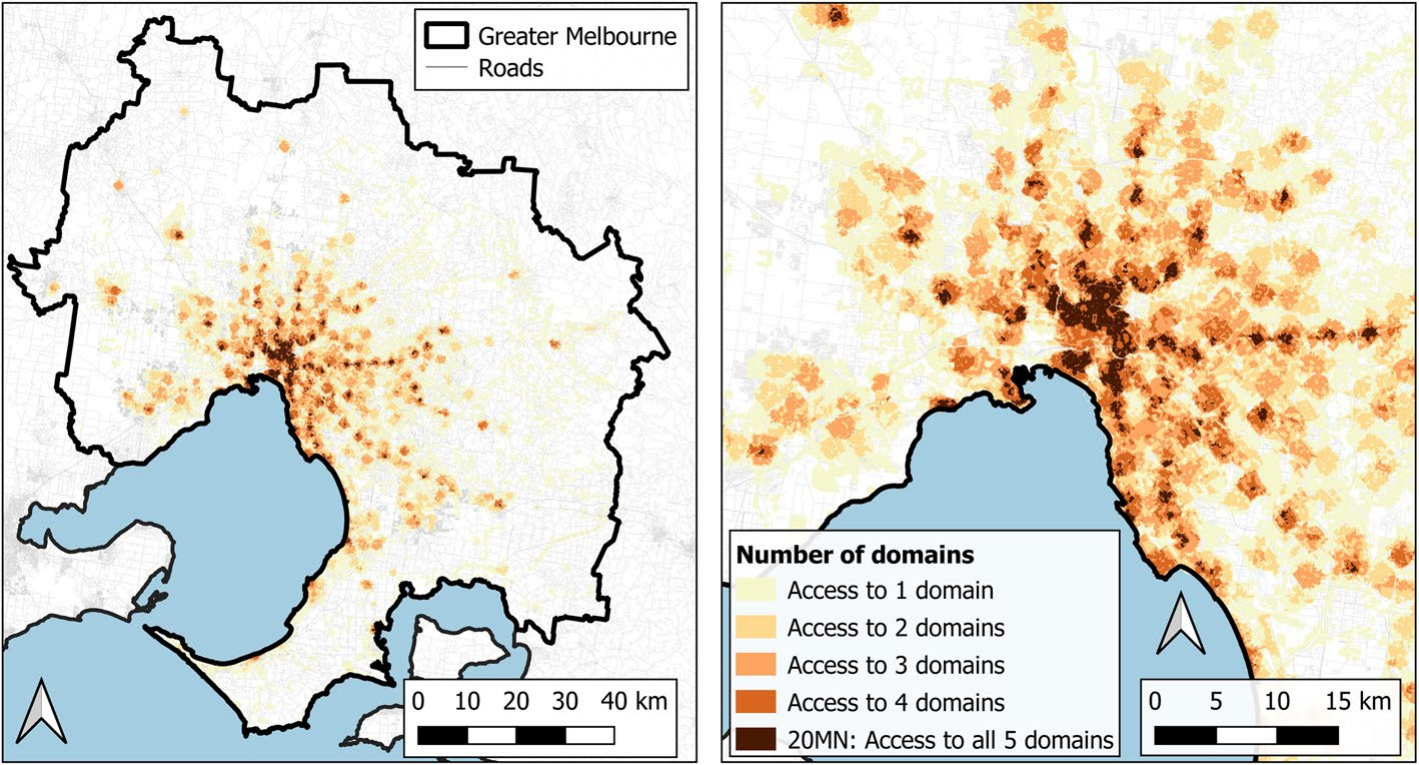
Count of domain access within Greater Melbourne (left) and the inner-mid Melbourne region (right)

**Fig. 5:**
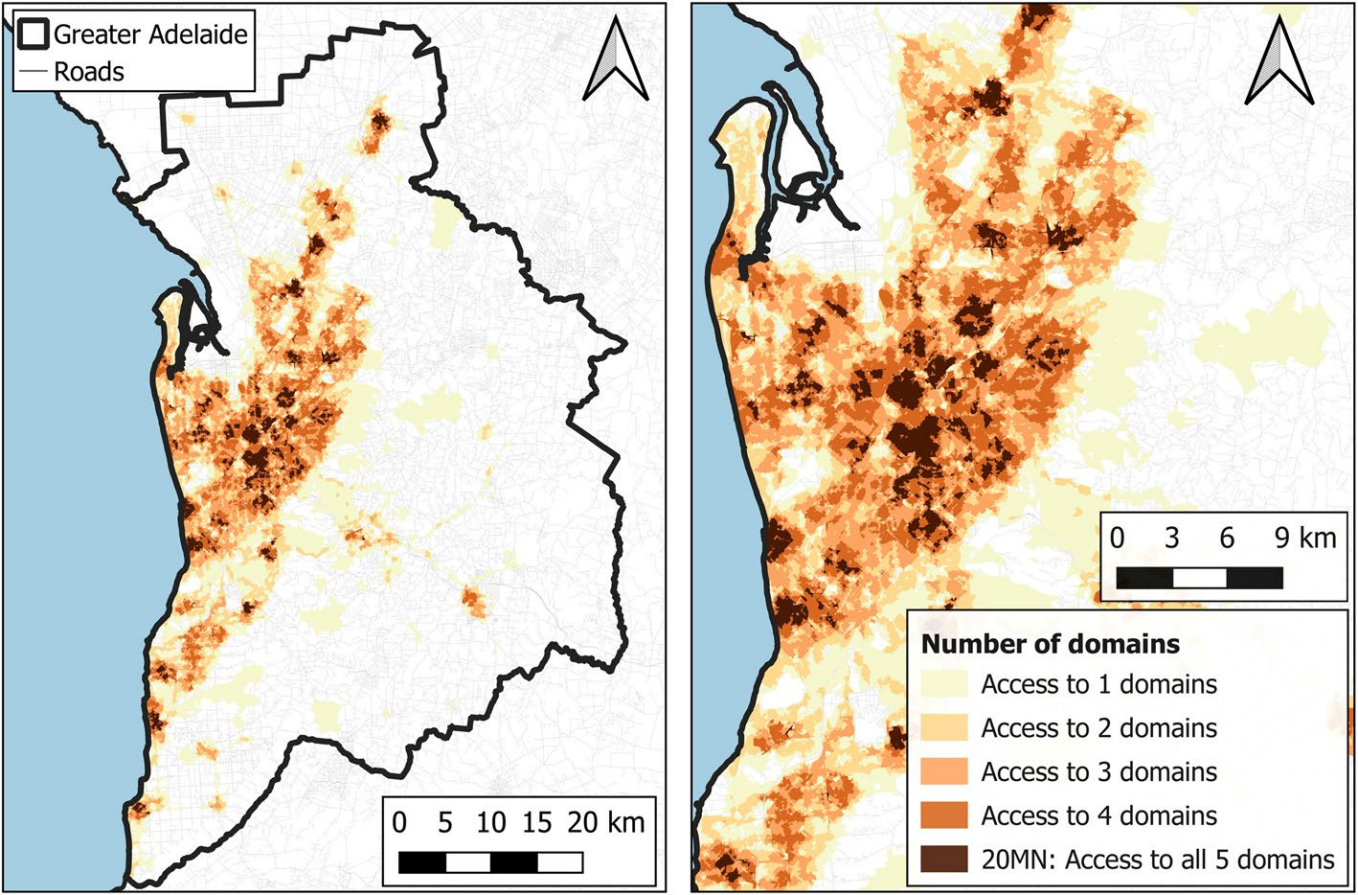
Count of domain access within Greater Adelaide (left) and the inner-mid Adelaide region (right)

Areas with access to each of the five domains and therefore a 20MN are represented in Fig. 4 for Melbourne and Fig. 5 for Adelaide. As expected, more areas considered to be 20MNs were clustered nearer to the city centres whilst in Melbourne there was also a noticeable pattern of areas with 20MNs appearing around the train stations in the mid and outer suburbs. Figures 4 and 5 also show the intensity of domain access across the two cities.

### Population and dwelling density by level of domain access

Population and dwelling counts were determined using data from the Australian Bureau of Statistics (ABS) 2016 census mesh block (the smallest geographical area defined by the ABS [48]). Mesh block centroids were used to determine how many domains that mesh block had access to. The ABS assign the dominant land use to each mesh block (e.g., residential, commercial, primary production, parkland). Over 95% of the population in both cities reside within mesh blocks categorised as residential. The total population with access to each domain was extracted (Table 3) in addition to the population density and dwelling densities of residential mesh blocks by the count of domains the mesh block had access to (Table 4).

**Table 3:** Population at the 2016 Census with access to each domain and the 20-minute neighbourhood

	Melbourne	Adelaide
	n.	n.
**Total population**	4,495,233	1,295,649
**Population with access to each domain**	**% of total population**	**% of total population**
- Healthy food	54.6%	51.1%
- Recreation resources	45.0%	56.4%
- Community resources	20.1%	17.8%
- Public open space	73.2%	76.1%
- Public transport	13.4%	64.3%
**Population with access to different number of domains**	**% of total population**	**% of total population**
- Access to no domains	9.8%	6.1%
- Access to 1 domain	30.4%	13.4%
- Access to 2 domains	25.5%	25.2%
- Access to 3 domains	18.0%	27.1%
- Access to 4 domains	10.8%	20.7%
- 20MN: Access to each of the 5 domains	5.5%	7.6%

**Table 4:** Population and dwelling density at the 2016 Census within mesh blocks classified as residential by count of domain access

	Melbourne (***n*** = 43,398 residential mesh blocks)	Adelaide (***n*** = 14,397 residential mesh blocks)
	Median (25th; 75th percentiles)	Median (25th; 75th percentiles)
**Population per square kilometre**	3386 (2589, 4373)	2704 (2161, 4133)
- Access to no domains (*n* = 3518 mesh blocks)	2203 (715, 3243)	1416 (592, 2246)
- Access to 1 domain (*n* = 12,794)	3122 (2241, 3877)	2405 (1751, 3020)
- Access to 2 domains (*n* = 11,197)	3333 (2706, 4071)	2595 (2083, 3122)
- Access to 3 domains (*n* = 8565)	3580 (2857, 4626)	2761 (2272, 3303)
- Access to 4 domains (*n* = 4911)	4314 (3201, 6593)	2857 (2343, 3481)
- 20MN: Access to each of the 5 domains (*n* = 2413)	6429 (4375, 9266)	3062 (2404, 4133)
**Dwellings per square kilometre**	1300 (1013, 1739)	1154 (924, 1463)
Access to no domains (*n* = 3518)	863 (311, 1205)	554 (239, 919)
Access to 1 domain (*n* = 12,794)	1160 (872, 1394)	993 (703, 1213)
Access to 2 domains (*n* = 11,197)	1279 (1047, 1548)	1093 (885, 1317)
Access to 3 domains (*n* = 8565)	1449 (1162, 2054)	1189 (994, 1481)
Access to 4 domains (*n* = 4911)	1884 (1341, 3286)	1255 (1030, 1644)
20MN: Access to each of the 5 domains (*n* = 2413)	3211 (2008, 4923)	1440 (1072, 2222)

## Results

When examining domains individually, over 50% of the population in each city met the access criteria for the healthy food domain whilst over 70% had access to open space (Table 3). In Melbourne, 45% met the access criteria for the recreational resources domain compared to 56% in Adelaide. The percentage meeting the access criteria for the community resources layer was much lower in both cities (20% Melbourne; 18% Adelaide) as this domain required access to six separate layers. The main difference between cities was in the percentage of the population meeting the access criteria for the public transport domain. This was to be expected given the different access criteria applied in the two cities (i.e., those further than 5 km from the General Post Office in Melbourne required access to a train station) with just 13% of the Melbourne population meeting the requirements of this domain compared to 64% in Adelaide.

Most of the Melbourne population (66%) had access to two or fewer domains compared to 45% in Adelaide which again likely reflects the differences in the public transport access criteria. In Melbourne, 5.5% of the population met the access criteria for each of the five domains and therefore were considered to have a 20MN. In Adelaide, this percentage was slightly higher at 7.6%.

Noticeably, there was a trend in the population density and dwelling density results with the lowest median density in areas without access to any of the domains and the highest median density in areas with access to all five domains (Table 4). The median number of people per square kilometre with access to all five domains (a 20MN) was 6429 in Melbourne and 3062 in Adelaide. The median number of dwellings per square kilometre with access to all five domains was also higher in Melbourne (3211) compared to Adelaide (1440).

## Discussion

The 20MN continues to be promoted as having many projected benefits [22, 24–27, 49, 50]. However, without defining and operationalising the 20MN, it remains difficult to implement a 20MN, monitor the progress of 20MN initiatives, and quantify the benefits. There are various ways that a 20MN could be expressed differently to what is proposed here including, for example, the addition of further attributes deemed important for everyday living, altering the modes of travel and the corresponding distance of a 20-minute trip, and allowing for more refined gradations of access to attributes. We have presented the first steps to *flexibly* operationalise the 20MN concept without resorting to the typical approach of using predefined administrative units. This ensures access can be determined from individual address points. This is an advance over existing liveability indicators that utilise predefined administrative units that measure access to various attributes according to their presence, number, and distance from some referent (e.g. within the boundaries of the unit or the distance from the geographic centroid) [51, 52]. We view pre-defined administrative units that specify arbitrary boundaries (e.g., statistical area 1, census tract) as having little, if any, inherently meaningful correspondence to a resident’s lived environment [53].

In support of our methodology, we examined variations in how the 20MN concept has been presented across various iterations of Melbourne’s planning strategies [24–27] (Table 1) but also internationally [16, 17, 19, 22]. We concede the attributes and access definition can, and should, be debated and that no single prescriptive definition may suit all contexts. Yet, the simplistic definition conveyed in policy documents falls short of specifying which amenities and services should be located within 20-minutes. This lack of specificity prevents a clear capacity to evaluate targeted interventions to increase 20MNs in urban areas.

The attributes that we selected represent a broad range of everyday services and amenities that may be considered to promote environmental, social, economic and health benefits across various demographic groups. In defining these attributes, we note three issues. First, access to an attribute is defined by the presence of a single attribute within the specified distance rather than the concentration of that attribute, with the partial exception of open space where a minimum concentration of 8 ha was required. Second, the selection of attributes did not consider those that might be harmful for environmental, social, or health reasons (e.g., fast food chains, presence of major thoroughfares leading to greater traffic, congestion, and pollution) and thus we did not exclude areas where harmful features were co-located with the selected layers. Third, in the case of recreational resources, our measure was limited to gyms including municipal-run facilities that are sometimes termed leisure centres and are available to non-members. In the Australian context, gyms operate all year round and it is not unusual for gyms (especially municipal-run gyms) to include facilities such as swimming pools and classes for activities such as yoga, in addition to weights and cardio equipment. Gyms also cater to people of all levels of fitness and abilities. We avoided sport specific facilities such as tennis courts due to the more limited general appeal. Whilst the availability of gyms provides the opportunity for people to use them, we acknowledge we did not differentiate between municipal-run and commercially-operated gyms, with the latter restricted to members and thus only accessible to those that can afford the membership fees. A key benefit of our overall approach is that each of the domains can be modified through the addition or removal of attributes. They can also be tailored, such that there may be multiple ways to meet the domain criteria. For example, for the healthy food domain, access could be obtained from having access to either a large supermarket *or* a smaller supermarket *and* greengrocer whilst in Melbourne, we altered the public transport criteria for those further from the city.

Our measures of access for most layers (i.e., a 20-minute walk equating to ~ 1.5-km) was consistent with the 2015 Plan Melbourne Refresh [25], with the exception of public transport and public open space where the access measures were guided by the literature [39–41] and land use planning guidelines, as detailed in Table 2. The recent suggestion that the 20MN relates to an 800-m walking distance [24] appears unfeasible given the current population density levels of Australian cities and the fact only a small percentage of the population were found to meet the 20MN criteria when our longer distance criterion was applied. The 1.5-km distance used represents the absolute maximum distance to which a number of attributes are considered accessible yet many of these may be closer. Additionally, our measure is based on a walking distance however the distance equating to a 20-minute trip would clearly vary based on the mode of travel and travel route used [54–57] in addition to other factors such as the slope of the land and the presence and quality of sidewalks. As the Portland Plan background report notes, the 20MN term is not intended to convey a specific metric but to reflect neighbourhoods where people can walk a relatively short distance from home to their daily destinations and services [16]. Thus, we believe the distance used combined with the five data domains adequately captured areas within a walkable distance to a range of services and amenities that meet daily needs.

Our final 20MN measure was based on all domain conditions being met. Future approaches may classify attributes by their level of relative importance (e.g., compulsory, very important, desirable but not essential) and modify the measure that way. This would also allow for a broader set of attributes to be included that may include, for example, secondary schools, banks, and playgrounds. In Figs. 4 and 5, areas that meet the access criteria for fewer domains are identifiable and this type of information could be used as part of the monitoring and implementation process with regards to transitioning these areas to a 20MN. Perhaps more importantly, the combination of population density and the domain intensity maps allows for the identification of areas with relatively high populations and relatively low amenity and service provision as these are clearly more important priority areas from a neighbourhood renewal perspective.

The flexible approach can (and should) also be tailored to different contexts nationally and internationally. Context-specific measures are more important than standardised measures for understanding local influences where contexts may differ in many ways. Different locations and settings vary in terms of physical and social structures and consequently essential attributes relevant to local needs may be unaccounted for by applying a single consistent measure [12]. Indeed, Portland, USA [16] had previously defined a 20MN according to the concentration of grocery stores, other retailers (e.g., convenience stores, coffee shops, health and personal services), park access points, public elementary (primary) schools, and frequent public transport in addition to street connectivity, sidewalks and slope. In some cases, it may be desirable to add additional attributes (e.g., additional health and community services or childcare facilities), perhaps substituting at the expense of others that might be replaced, to ensure the area meets the everyday needs of the population of interest. Our Australian measure included access to health services such as general practitioners and pharmacies which, as Whitzman suggests [58], is important given Australia’s ageing population. In addition to the different contexts, there may be a desire to alter the attributes and distances and/or travel modes based on the population of interest (e.g., apply a shorter access distance in areas with a higher number of elderly people, or families with young children). An additional consideration related to travel, especially for the elderly and young children, could also include a safe walking environment indicator that consists of traffic calming features and safe crossing points, and perhaps even the slope of the land to avoid large hills.

Whilst Australia has a largely urban population, the population density in urban areas is often much lower than in other developed nations [59]. Increasing population density is seen as an important means of facilitating better access to amenities and services [7, 49, 60]. However, a recent report [61] forecasts significant challenges to the infrastructure of Australian cities (e.g., housing, traffic) and their ability to support rapid population growth whilst Neuman (2005) further discusses the potential negative implications of poorly managed density in his paper on the compact city fallacy [62]. In Adelaide, we found that 20MNs were achieved in neighbourhoods with lower population densities than in Melbourne. A likely explanation for this is that in Melbourne several high-rise apartment buildings have been approved in areas with high-service provision [34, 35, 63, 64]. This potentially means the higher population density of 20MNs in Melbourne has been achieved through locating more people near services rather than locating services near people. Further explanation is warranted on the role of high population densities in achieving the projected benefits of the 20MN or whether such benefits also hold true in less population dense areas such as those observed in Adelaide.

Whilst potential benefits exist through the development of local 20MNs, it is important to acknowledge and highlight the challenges of this concept [54, 58, 65, 66]. Although we were able to demonstrate the applicability of our approach across two cities, direct comparisons should be undertaken with care. This is, firstly, because we used a different public transport measure for areas further than five kilometres from the centre of the city in Melbourne. In this instance, we believe the criterion requiring train access outside of the inner Melbourne area is appropriate given the larger geographic size of this city and that commuters in Melbourne have a longer average commute distance [42]. An additional point of caution relates to the use of different data sources which was necessary in the absence of consistent national level data.

## Conclusion

The need for an operationalised measure of the 20MN is clear from both an implementation and evaluation perspective. This challenge was addressed in this study, conceding there is clearly more than one way to define a 20MN. Our approach provides the flexibility to contextually tailor key attributes and access measures. It is hoped that modified versions of this measure can be applied to other cities nationally and internationally to determine the presence of and need for 20MNs. By applying our operationalised measure to two Australian cities, our analysis indicates that only a small percentage of the population live in what we would consider to be a 20MN. This measure can now be used to assess the projected benefits of the 20MN including whether they promote healthy and local living.

## Supplementary Information

**Additional file 1.** Data sources table.

## Data Availability

The datasets used for the during the current study are available from the corresponding author on reasonable request.

## Abbreviation

20MN: 20 minute neighbourhood
