# Supplementary material for: Operationalising the 20-minute neighbourhood
Source: Int J Behav Nutr Phys Act. 2022 Feb 12;19:15. doi: 10.1186/s12966-021-01243-3 (PMC8841074; doi:10.1186/s12966-021-01243-3)
Supplement: Supplementary file 1 — Additional file 1. Data sources table. [file 12966_2021_1243_MOESM1_ESM.docx]

**Additional File 1: Data sources table**

| **Spatial extent** | Australian Bureau of Statistics Greater Capital City Statistical Areas  [https://www.abs.gov.au/ausstats/abs@.nsf/Lookup/by%20Subject/ 1270.0.55.001~July%202016~Main%20Features~Greater%20 Capital%20City%20Statistical%20Areas%20(GCCSA)~10003](https://www.abs.gov.au/ausstats/abs@.nsf/Lookup/by%20Subject/%201270.0.55.001~July%202016~Main%20Features~Greater%20%20Capital%20City%20Statistical%20Areas%20(GCCSA)~10003) |
| --- | --- |
| **Mesh blocks** | Australian Bureau of Statistics Australian Statistical Geography Standard (ASGS)  <https://www.abs.gov.au/websitedbs/d3310114.nsf/home/australian+statistical+geography+standard+(asgs)>  Australian Statistical Geography Standard (ASGS): Volume 1 - Main Structure and Greater Capital City Statistical Areas  <https://www.abs.gov.au/ausstats/abs@.nsf/Lookup/by%20Subject/1270.0.55.001~July%202016~Main%20Features~Mesh%20Blocks%20(MB)~10012> |
| **Address points - Melbourne** | Department of Environment, Land, Water & Planning Road Network - VicMap Address  <https://www.land.vic.gov.au/maps-and-spatial/spatial-data/vicmap-catalogue/vicmap-address#:~:text=Vicmap%20Address%20is%20the%20authoritative,and%20custodian%20of%20property%20addresses> |
| **Address points - Adelaide** | Department of Planning, Transport and Infrastructure, Land Services Group |
| **Roads - Melbourne** | Department of Environment, Land, Water & Planning Road Network - Vicmap Transport  <https://discover.data.vic.gov.au/dataset/road-network-vicmap-transport> |
| **Roads - Adelaide** | Department of Planning, Transport and Infrastructure Statewide Road Network  [https://data.sa.gov.au/data /dataset/roads](https://data.sa.gov.au/data%20/dataset/roads) |
| **Large supermarkets, small supermarkets and fruit and vegetable store – Melbourne and Adelaide** | The food category is composed of large (main) supermarkets, small supermarkets, fruit and vegetable outlets commonly referred to as greengrocers. The data was obtained from Google radar search. A sub-selection of the corresponding results were refined through ’keyword’ and ‘type’ attribute selections. Generally, the obtained raw data was screened and further selection criteria, mainly based on attributes ‘name’ and types (‘type1’, ‘type2’, etc.), manually identified. Within the Place API, Google assigned a defined set of type categories for each place. As additional refinement of ‘supermarket’, ‘fruit’ and ‘vegetable’ data was achieved through manual exclusion of establishment based on recorded names in the search result.  Main and small supermarkets were derived from the same Google search results based on the keyword ‘supermarket’, features marked as ‘permanently closed’ in the Google search results were excluded.   - Main supermarkets were identified through names such as ‘Coles’ and ‘Woolworth’ in combination with excluding unwanted facilities within this selection set representing smaller outlets, such as ‘Metro’ or ‘Express’ - The small supermarket data was obtained through the names such as “Aldi’, ‘IGA’, ’Metro’ ‘Foodland’, excluding names like ‘Express’ or ‘Caltex’, and excluding type values such as ‘bakery', 'convenience_store', 'liquor_store', 'restaurant', etc. - The information on greengrocers was sourced from the two separate Google Radar searches using keywords ‘fruit’ and ‘vegetable’, respectively. The final selection of data was then merged (unioned). - The selection of the ‘fruit’ data was based on finding substrings within the ‘name’ such as ‘fruit', 'fresh', 'green', 'grocer', 'organic', 'veg’ and matching type categories such as ‘store’, ‘food’, ’grocery_or_supermarket’, ‘supermarket’ excluding names like ‘juice’, ‘butcher', 'smoothie'. - Subsetting the search using the keyword ’veg’ as an addition to achieving a greengrocers outlet locality, involved selection and exclusion of similar type and ‘name’ values as in ‘fruit’.   Identification of attributes: Defining keywords for use in Google Places API search   - Google Radar Search1: Search of up to 200 places based on x/y point location – max. search radius 50km; - 3km radius used as conservative measure retrieving all relevant attribute/category types; The Radar Search returns two main objects: Place-Id and a location x/y (longitude/latitude) - Retrieving further location details using Google Place Details2 search: Radar Search derived Place-Id utilised to request further details such as Name, formatted address, category types3 (several, generally around 5 to 6 types– potentially in order of relevance), daily opening times and a boolean attribute whether the establishment is permanently closed. The category types can be used for the identification of industry type assignment and used as estimates. - Searches are automated using Python scripting – each record is stored as a delimited line in text files (csv), or inserted into a database - Data can be mapped using x/y point information   1 Google retired Radar Search in June 2018; <https://cloud.google.com/blog/products/maps-platform/announcing-deprecation-of-place-add>; formerly under: <https://developers.google.com/maps/documentation/javascript/places#radar_search_requests>  2 <https://developers.google.com/places/web-service/details>  3 <https://developers.google.com/places/web-service/supported_types> |
| **Primary school - Melbourne** | Victorian State Government Department of Education and Training: School Locations 2017  <https://discover.data.vic.gov.au/dataset/school-locations-time-series>  A subset was applied through setting the attribute ‘Subtype_Name’ to 'Primary Schools' |
| **Primary school - Adelaide** | South Australian Government Education and Child Development site  <https://data.sa.gov.au/data/dataset/child-development-sites> |
| **General practitioner (GP)** | National data, Health-Direct, sourced via AURIN  <https://data.aurin.org.au/dataset/healthdirect-nhsd-point-december-2017-na>  Locational public health information on general practitioners/doctors and pharmacies was obtained via a special request to AURIN on the National Health Services Directory (NSHD), which was delivered in 30 November 2017. A further NSHD update was received through AURIN on 3rd January 2018. The NSHD is a nation-wide health services data directory managed and compiled by the non-profit government organisation health-direct as data custodian and owner.  The final dataset was generated through an attribute query ("ServiceT_1" = 'General Practice/GP (doctor)') of the NSHD information. |
| **Pharmacy** | National data, Health-Direct, sourced via AURIN  <https://data.aurin.org.au/dataset/healthdirect-nhsd-point-december-2017-na>  The pharmacy locational data was obtained from NSHD data via AURIN using a subset (based on "ServiceT_1" = 'Pharmacy'). The data was derived from the same data delivery (30th Nov 2017) and updated using the latest NSHD updates (1st Jan 2018). |
| **Library - Melbourne** | Library data sourced from the PSMA^1^ locality data (‘Features of Interest 2016’) obtained from the AURIN online portal. A subset was of the PSMA data was generated through the attribute query: 5feature_c = ’LIBRARY’, and spatially refined to state of Victoria.  ^1^PSMA was a national, authoritative location data provider – since Oct 2020 it is trading under Geoscape, <https://geoscape.com.au/> |
| **Library - Adelaide** | SA_Govt_LGASA  Locations and Attributes of 174 Public Libraries in South Australia, sourced via AURIN.  Libraries in PSMA for Adelaide were not available |
| **Post office - Melbourne** | Post office locations, also obtained via the AURIN portal, were created subsetting PSMA ‘Features of Interest 2016’ (based on attribute “5feature_c” = ’POST OFFICE’), spatially refined to the extent of the state of Victoria |
| **Post office - Adelaide** | Google nearby-search 2018;  type1 = ‘postoffice or store’ ;  manual filter– store names identifiable as post offices (6 only);  Post offices in PSMA for Adelaide were not available |
| **Café – Melbourne & Adelaide** | Google Radar Search  Location data for cafés was retrieved on 26^th^ Nov 2017 via the Google Radar search using the keyword “café”. The data was examined and further refined based on Google’s type classes reflecting industry type classifications, naming of the establishment, opening times, permanently closed assignment. A primary query formulated was based on Google’s assigned types (type1 including 'cafe'; and type2 including 'bakery', 'bar', 'food', 'meal_takeaway', 'restaurant'; but type2 excluding 'bar' and type3 excluding 'liquor_store', 'lodging', 'night_club’; in addition, the name attribute excluding 'Oporto'). Further exclusion was conducted through manual selection of name records identifying apparent unsuitable descriptions. |
| **Gym - Melbourne** | Victorian State Government Department of Health and Human Services  <https://discover.data.vic.gov.au/dataset/sport-and-recreational-facilities>  The selection of ‘Sport and recreational facilities’ data was conducted refining the category 'SportsPlay' to the value of ‘Fitness/Gymnasium Workouts’. Manual refinement of the extracted list took place, excluding other types of sport and fitness places. |
| **Gym - Adelaide** | Google radar-search 2018;  type1 = ‘gym’ and type2=’health’ and name not in a list of unsuitable places, such as ‘karate’,’aikido’, ’taekwondo’ ‘soccer’, ‘dance’, ‘massage’, ‘pilates’, ‘football’, yoga. Further manual refinement of the extracted list took place, and 185 place names were excluded from the search result. |
| **Public open space - Melbourne** | Victorian Planning Authority Open Space  <https://discover.data.vic.gov.au/dataset/open-space>  Victorian Planning Authority Public Open Space - 400m Walkable Catchment  <https://discover.data.vic.gov.au/dataset/public-open-space-400m-walkable-catchment> |
| **Public open space - Adelaide** | Sourced from previous study involving one of this paper’s authors: Daker, M., et al. (2016). "Validating and measuring public open space is not a walk in the park." Australian Planner **53**(2): 143-151. |
| **Public transport - Melbourne** | Public Transport Victoria bus stops  <https://discover.data.vic.gov.au/dataset/ptv-metro-bus-stops>  Public Transport Victoria tram stops  <https://discover.data.vic.gov.au/dataset/ptv-metro-tram-stops>  Public Transport Victoria trains stations  <https://discover.data.vic.gov.au/dataset/ptv-metro-train-stations> |
| **Public transport - Adelaide** | Adelaide metro-stops and tram-stops:  <http://www.adelaidemetro.com.au> via [data.sa.gov.au](http://www.data.sa.gov.au) |
